# Rotigotine transdermal system for long-term treatment of patients with advanced Parkinson’s disease: results of two open-label extension studies, CLEOPATRA-PD and PREFER

**DOI:** 10.1007/s00702-012-0925-5

**Published:** 2012-12-04

**Authors:** Peter A. LeWitt, Babak Boroojerdi, Erwin Surmann, Werner Poewe

**Affiliations:** 1 Department of Neurology, Henry Ford Hospital, Detroit, MI USA; 2 Department of Neurology, Wayne State University School of Medicine, Detroit, MI USA; 3 UCB Pharma, Raleigh, NC USA; 4 UCB Pharma, Monheim, Germany; 5 Medical University of Innsbruck, Innsbruck, Austria

**Keywords:** Rotigotine transdermal system, Open-label, Clinical trial, Parkinson’s disease

## Abstract

Open-label extensions [studies SP516 (NCT00501969) and SP715 (NCT00594386)] of the CLEOPATRA-PD and PREFER studies were conducted to evaluate the safety, tolerability and efficacy of the dopaminergic agonist, rotigotine, over several years of follow-up in patients with advanced Parkinson’s disease (PD). Eligible subjects completing the double-blind trials received open-label adjunctive rotigotine (≤16 mg/24 h) for up to 4 and 6 years in Studies SP516 and SP715, respectively. Safety and tolerability were assessed using adverse events, vital signs and laboratory parameters, and efficacy assessed using the unified Parkinson’s disease rating scale (UPDRS). Of the 395 and 258 patients enrolled in the SP516 and SP715 studies, 48 and 45 % completed, respectively. Adverse events were typically dopaminergic effects [e.g., somnolence (18–25 %/patient-year), insomnia (5–7 %/patient-year), dyskinesias (4–8 %/patient-year) and hallucinations (4–8 %/patient-year)], or related to the transdermal application of a patch (application site reactions: 14–15 %/patient-year). There were no clinically relevant changes in vital signs or laboratory parameters in either study. Mean UPDRS part II (activities of daily living) and part III (motor function) total scores improved from double-blind baseline during dose titration, then gradually declined over the maintenance period. In study SP516, mean UPDRS part II and III total scores were 0.8 points above and 2.8 points below double-blind baseline, respectively, at end of treatment. In study SP715, mean UPDRS part II and III total scores were 4.1 points above and 0.2 points below baseline, respectively, at end of treatment. In these open-label studies, adjunctive rotigotine was efficacious with an acceptable safety and tolerability profile in patients with advanced PD for up to 6 years.

## Introduction

Levodopa (l-dopa), an amino acid precursor of dopamine, has been the mainstay of therapy for Parkinson’s disease (PD) for more than 40 years (LeWitt 2008). However, long-term treatment with l-dopa is often associated with the development of motor response fluctuations and dyskinesias (Nutt 2001). These problems are intrinsic to l-dopa therapy and are possibly the result of the pulsatile dopaminergic agonist receptor stimulation that is associated with the short peripheral clearance half-life of l-dopa (Olanow et al. 2006; Antonini et al. 2009). Research for new PD therapies has focused on developing drugs with extended dopaminergic stimulation, particularly dopaminergic agonists (LeWitt 2010). The latest dopaminergic agonist to be marketed is rotigotine, a non-ergolinic dopamine receptor agonist with activity across D1 through D5 receptors, as well as at selected adrenergic and serotonergic sites. Rotigotine is administered via a transdermal delivery system left in place for 24 h. This enables continuous drug delivery and, therefore, stable plasma drug concentrations over the 24-h period (Jenner 2005; Rascol and Perez-Lloret 2009; Boroojerdi et al. 2010). Rotigotine transdermal system is licensed by the US Food and Drug Administration for the treatment of the signs and symptoms of early-stage idiopathic PD, and is approved for the treatment of early- and advanced-stage idiopathic PD, and for moderate-to-severe idiopathic restless legs syndrome in adults in the European Union.

In several short-term, randomized, double-blind clinical studies, rotigotine has demonstrated statistically significant treatment benefits and good tolerability in both early- and advanced-stage PD (Parkinson Study Group 2003; Giladi et al. 2007; Jankovic et al. 2007; Watts et al. 2007). Like other dopaminergic agonists, it can be used as a substitute for the previous dose of l-dopa needed by patients with either early- or advanced PD (Pham and Nogid 2008). In the RECOVER (randomized evaluation of the 24-h coverage: efficacy of rotigotine) study, rotigotine, added to l-dopa, was associated with significant improvements in early-morning motor function and nocturnal sleep disturbances in patients with early-morning motor dysfunction (Trenkwalder et al. 2011). Other clinical outcomes with rotigotine have included improvement in overall performance in activities of daily living (ADLs) and a reduction in the proportion of l-dopa-treated patients awakening in an “OFF” state (Pahwa et al. 2009). In addition, adjunctive rotigotine resulted in significant reductions in “OFF” time and was generally well tolerated in l-dopa-treated patients with advanced PD with motor fluctuations in two multicenter, 6-month, randomized, double-blind, placebo-controlled trials—CLEOPATRA-PD (clinical efficacy of Pramipexole and transdermal rotigotine in advanced PD) (Poewe et al. 2007) and PREFER (prospective randomized evaluation of a new formulation: efficacy of rotigotine) (LeWitt et al. 2007).

As PD progresses, patients tend to have increased symptomatology and disability. Furthermore, patients with advanced PD require increasing doses of l-dopa, resulting in more frequent drug-induced problems such as dyskinesias and motor fluctuations. Only a few open-label studies have been conducted that evaluate the long-term safety and efficacy of the available non-ergot dopaminergic agonists in patients with PD (Rascol et al. 2000; Holloway et al. 2004; Hauser et al. 2007; Parkinson Study Group CALM Cohort Investigators 2009). Here, we report the results of open-label extensions of the CLEOPATRA-PD (study SP516; clinicaltrials.gov identifier: NCT00501969) and PREFER (SP715; clinicaltrials.gov identifier: NCT00594386) studies, conducted to evaluate the long-term safety, tolerability and efficacy of adjunctive transdermal rotigotine in patients with advanced PD.

## Materials and methods

### Patients

Eligibility criteria for the PREFER and CLEOPATRA-PD double-blind studies have been reported previously (Poewe et al. 2007; LeWitt et al. 2007). These were, in brief, a diagnosis of idiopathic PD for at least 3 years, an average of 2.5 h of “OFF” time on the 24-h self-report motor function diaries, and Hoehn and Yahr stage II to IV in both “ON” and “OFF” states. In addition, patients had to be receiving stable doses of l-dopa of ≥200 mg/day in at least twice daily doses for entry to PREFER and ≥300 mg/day in at least three daily doses for entry to CLEOPATRA-PD, with no change in any concomitant anti-PD medication for at least 28 days prior to baseline. Patients were excluded from the double-blind studies if they had received therapy with a dopaminergic agonist, methylphenidate, amphetamine, entacapone or tolcapone within 28 days of baseline.

Patients who completed the double-blind dose maintenance period of their study were eligible for entry into the respective open-label extension, provided there were no ongoing serious adverse events (AEs) related to trial medication. Those receiving approved concomitant PD medications were required to be on a stable dose which remained unchanged until the dose titration period with rotigotine was complete and the optimal dose of rotigotine had been confirmed [after 1 month of maintenance (see below)]. In addition, investigators were encouraged not to increase the doses of other PD medications or to initiate other adjunctive PD therapy during the maintenance period until the rotigotine dose was at its maximum of 16 mg/24 h. During the open-label studies, use of l-dopa (in combination with benserazide or carbidopa) was continued and the following medications were also permitted: selegiline, rasagiline, anti-cholinergic drugs, entacapone, tolcapone, certain atypical neuroleptics (olanzapine, ziprasidone, aripiprazole, clozapine, quetiapine) and modafinil. Anti-emetic drugs were also permitted to treat nausea and vomiting caused by excess dopaminergic stimulation.

### Study design

**Fig. 1:**
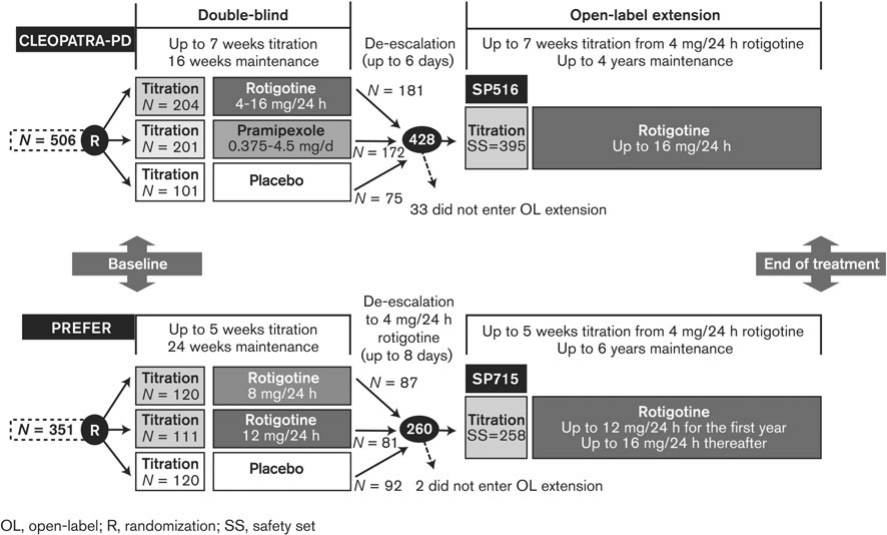
Design of open-label extension studies for participants from the CLEOPATRA-PD and PREFER studies

At the end of the double-blind studies, rotigotine-treated patients who decided to participate in the extensions had their dose of rotigotine de-escalated in a blinded fashion to 4 mg/24 h over either a 6-day period (study SP516; the CLEOPATRA-PD extension study) or an 8-day period (study SP715; the PREFER extension study) (Fig. 1). Patients who completed the double-blind studies on a dose of 4 mg/24 h did not require dose de-escalation and patients who had been randomized to placebo continued to receive placebo during de-escalation. Dose de-escalation was followed by up-titration in 2 mg/24 h increments every 7 (±3) days to the subject’s optimal dose (up to a maximum of 16 mg/24 h in both studies except for the first year of study SP715 when the maximum dose was 12 mg/24 h). The titration periods lasted up to 7 weeks. If AEs thought to be related to excessive dopaminergic stimulation occurred during the titration period, reduction of the rotigotine dose was permitted once during this period. Once the titration period was complete, or the optimal dose had been reached, the maintenance period began. Rotigotine dose could be increased or decreased as needed during the maintenance period to maintain an effective dose for each patient. End of rotigotine treatment could have occurred at any time during the trials. At the time of study closure, which was after up to 4 years in study SP516 and up to 6 years in study SP715, an end of treatment (EoT) visit took place, at which patients began dose de-escalation in 2 mg/24 h steps every 2 days over a period of up to 12 days. The end of treatment visit was also conducted for patients who withdrew prematurely, provided that the assessments could be performed within 24 h of the final patch administration. A safety follow-up visit was conducted within 28 days of the final patch application. Clinic visits occurred at the start of the maintenance period, 1 and 3 months later, and then at 3-month intervals until the end of the maintenance period.

### Outcome measures

The primary variables were AEs, as reported spontaneously by the patient or observed by the investigator; and change from baseline in vital signs, body weight, electrocardiogram, clinical laboratory values, Epworth sleepiness scale (ESS) scores, and physical and neurological examinations over the course of the study.

Efficacy outcome measures in both studies were the unified Parkinson’s disease rating scale (UPDRS) part II (ADLs) and part III (motor examination), and Hoehn and Yahr assessments (to assess the severity and progression of disease). UPDRS part IV (complications of therapy) was used for the assessment of the incidence of dyskinesias (“What proportion of the waking day are dyskinesias present?”) and the duration of “OFF” time (“What proportion of the waking day is the patient “OFF” on average?”).

UPDRS parts II and III, and Hoehn and Yahr staging were completed while the patient was in an “ON” state. All UPDRS assessments were performed, and the investigator’s clinical global impression (CGI) of the patient’s symptoms recorded, at every clinic visit. Hoehn and Yahr staging was recorded in the “ON” state at visits 11 and 13 of study SP516, and visit 9 of study SP715; then repeated every 6 months throughout the treatment period and at the EoT visit for both studies. Changes in adjunctive l-dopa therapy were monitored throughout both studies.

### Data analysis

Safety and efficacy analyses were performed on the safety set—defined as all subjects who received at least one dose of rotigotine in the open-label extension—and are reported as observed cases. In addition, the UPDRS part IV item “What proportion of the waking day is the patient ‘OFF’ on average?” was analyzed based on last observation carried forward values. The primary safety variables were analyzed descriptively. AEs were evaluated according to their seriousness, intensity, outcome and causality. A serious AE was one which, at any dose, was fatal and life-threatening, resulted in persistent or significant disability or incapacity, resulted in hospitalization or the prolongation of existing hospitalization, or was considered to be an important medical event. The intensity of each AE was classified as mild (did not interfere with routine activities), moderate (interfered with routine activities) or severe (subject was unable to perform routine activities). The outcome of each AE was described as fatal, ongoing, recovering, recovering with sequelae, recovered or lost to follow-up. The causality of each AE was related to the likelihood of a relationship with the study drug and ranged from “not related” to “highly probable”. An exposure-adjusted incidence of AEs was calculated by taking the total number of events reported (both unique and non-unique) and dividing it by the sum, over all patients reporting that particular AE, of the treatment period in years, yielding an AE incidence per patient-year.

Endpoints were changed from baseline to EoT in each outcome measure where baseline was visit 2 of the relevant double-blind trial except for Hoehn and Yahr, where baseline was visit 1 of the double-blind study, and the UPDRS part IV item “What proportion of the waking day are dyskinesias present?” in study SP715 where baseline was visit 1 of the open-label study.

Descriptive statistics were provided for measured values and changes from baseline by visit for the UPDRS parts II and III. A responder analysis for UPDRS parts II and III was performed, where a responder was defined as a subject who had improved by ≥20 % in the UPDRS parts II and III sum score compared with baseline.

## Results

### Patient disposition and treatment

Of the 506 patients randomized in the CLEOPATRA-PD study, 428 (85 %) completed and were eligible to enroll in the open-label extension (study SP516; Fig. 1) (Poewe et al. 2007). Of these, 395 did so, with 189 (48 %) still participating in the study at study closure. Of the 351 patients randomized in the PREFER study, 260 (74 %) completed, with 259 eligible to enroll in the open-label extension (study SP715; Fig. 1; LeWitt et al. 2007). All but one patient did so, with 115 (45 %) still participating at study end. Demographic and clinical characteristics at double-blind baseline were similar for subjects in both open-label extension studies (Table 1), with the majority of subjects (55 % in study SP516 and 59 % in study SP715) having a CGI of four at baseline, indicative of moderately severe PD.

**Table 1:** Demographic and clinical characteristics at double-blind baseline (safety set, both studies)

Parameter	Study SP516 (*N* = 395)	Study SP715 (*N* = 258)
Age in years, mean ± SD (range)	64.4 ± 9.2 (39–84)	66.4 ± 9.6 (34–88)
Male, *N* (%)	251 (64)	173 (67)
Caucasian, *N* (%)	385 (97)	240 (93)
Time since first diagnosis in years, mean ± SD (range)	8.5 ± 4.6 (3–29)	7. 8 ± 4.5 (2–24)
UPDRS part II score in years, mean ± SD (range)	12.3 ± 5.9 (0–33)	12.6 ± 6.4 (0–36)
UPDRS part III score in yeras, mean ± SD (range)	27.0 ± 11.7 (1–65)	26.1 ± 13.8 (0–83)

*SD* standard deviation, *UPDRS* unified Parkinson’s disease rating scale

**Fig. 2:**
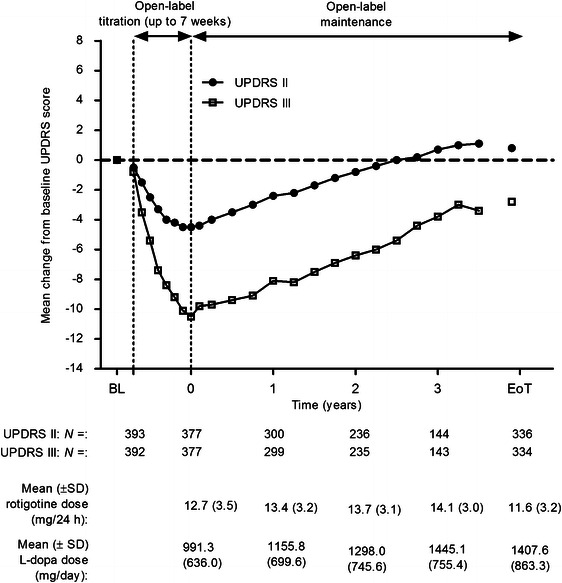
Change from baseline (visit two of double-blind study) in UPDRS part II (ADL) and part III (motor function) scores during open-label treatment in study SP516; safety set, observed cases. Mean rotigotine and l-dopa doses shown by timepoint. *ADL* activities of daily living, *BL* double-blind baseline, *EoT* end of treatment, *l-* *dopa* levodopa, *SD* standard deviation, *UPDRS* united Parkinson’s disease rating scale. Patients were followed for up to 4 years; data are not shown beyond 3.5 years due to <50 patients with measurements at these timepoints

**Fig. 3:**
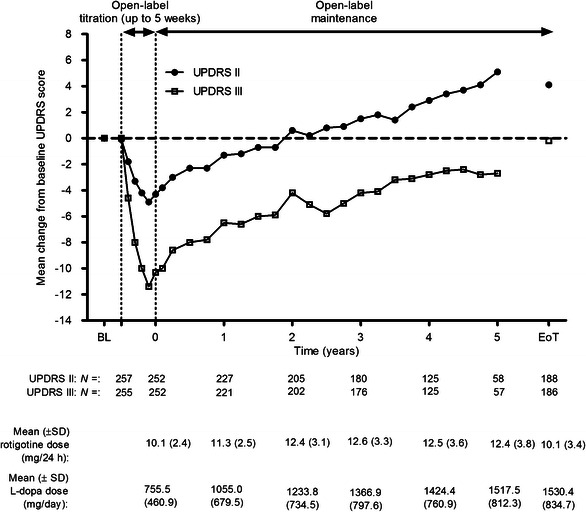
Change from baseline (visit two of double-blind study) UPDRS part II (ADL) and part III (motor function) scores during open-label treatment in SP715; safety set, observed cases. Mean rotigotine and l-dopa doses shown by timepoint. *ADL* activities of daily living, *BL* double-blind baseline, *EoT* end of treatment, *l-* *dopa* levodopa, *SD* standard deviation, *UPDRS* united Parkinson’s disease rating scale. Patients were followed for up to 4 years; data are not shown beyond 3.5 years due to <50 patients with measurements at these timepoints

In study SP516, the most common rotigotine dose on entering the open-label maintenance phase was the 16 mg/24 h dose (41 % of patients) while for study SP715, it was the 12 mg/24 h dose (54 % of patients). The rotigotine dose remained relatively stable over the maintenance periods of both studies (see annotated doses on Figs. 2, 3) with a slightly higher mean dose at EoT in study SP516 (11.6 ± 3.2 mg/24 h) compared with study SP715 (10.1 ± 3.4 mg/24 h). Mean exposure to rotigotine in studies SP516 and SP715 (double-blind and open-label phases combined) was 1,017.9 (±458.1) days and 1,538.8 (±609.3) days, respectively. l-Dopa was taken concomitantly by all subjects during the treatment periods of both studies and its mean daily dose increased over time in both (see annotated doses on Figs. 2, 3).

Other anti-PD drugs taken during the treatment period included selegiline and rasagiline (18 % of patients in study SP516 and 28 % in study SP715), amantadine (26 % of patients in study SP516 and 27 % in study SP715) and other dopamine agonists (9 % in study SP516 and 15 % in study SP715). Anti-emetics and anti-nauseants were taken by 11 patients (3 %) during the treatment period of study SP516 and by 28 patients (11 %) during the treatment period of study SP715.

### Safety and tolerability

In study SP516, 90 % of patients reported at least one AE and the figure was 100 % in study SP715. For both studies, the absolute and exposure-adjusted incidence of those AEs reported with an incidence ≥5 % per patient-year during open-label treatment is shown in Table 2. Data on AE severity and its link to study discontinuation are also shown. Most AEs were mild or moderate in intensity; only 8 and 9 % of all AEs reported in studies SP516 and SP715, respectively, were recorded as severe and few resulted in study discontinuation. In addition, most AEs (63 % in study SP516 and 62 % in study SP715) had resolved at the end of trial participation.

**Table 2:** AEs reported with an absolute incidence ≥5 % per patient-year during open-label treatment in studies SP516 and SP715 (absolute and exposure-adjusted incidence, number leading to discontinuation and severity; safety set, both studies)

	Study SP516 (*N* = 395)					Study SP715 (*N* = 258)				
	No. of events	No. of patients (%)	Exposure-adjusted incidence (percentage per patient-year)	No. of events leading to discontinuation/no. (%) patients discontinued	Severity (%) mild and severe^a^	No. of events	No. of patients (%)	Exposure-adjusted incidence (percentage per patient-year)	No. of events leading to discontinuation/no. of (%) patients discontinued	Severity (%) mild and severe^a^
Any AE	2,798	357 (90.4)	293.1	93/79 (20.0)	55.5 % mild	4,653	258 (100)	482.6	88/72 (28)	51.7 % mild
										9.4 % severe^b^
					7.8 % severe					
Preferred term										
Somnolence	170	133 (33.7)	18.3	2/2 (0.5)	75.9 % mild	236	150 (58.1)	24.9	3/3 (1.2)	68.6 % mild
										1.7 % severe
					2.4 % severe					
Fall	121	63 (15.9)	12.9	None	52.9 % mild	212	104 (40.3)	22.2	2/2 (0.8)	47.6 % mild
										6.6 % severe
					17.4 % severe					
ASRs^c^	138	103 (26.1)	14.9	14/14 (3.5)	77.5 % mild	130	84 (32.6)	13.8	1/1 (0.4)	83.1 % mild
										0 % severe
					5.1 % severe					
Peripheral edema	35	33 (8.4)	3.7	None	68.6 % mild	112	80 (31.0)	11.7	1/1 (0.4)	67.0 % mild
										3.6 % severe
					None severe					
Urinary tract infection	51	32 (8.1)	5.4	1/1 (0.3)	52.9 % mild	95	58 (22.5)	9.9	None	40.0 % mild
										10.5 % severe
					2.0 % severe					
Nausea	62	54 (13.7)	6.7	2/2 (0.5)	82.3 % mild	94	61 (23.6)	9.5	None	63.8 % mild
										4.3 % severe
					3.2 % severe					
Arthralgia	40	34 (8.6)	4.3	None	45.0 % mild	80	61 (23.6)	8.3	None	47.5 % mild
										5.0 % severe
					7.5 % severe					
Hallucination	39	35 (8.9)	4.2	6/5 (1.3)	48.7 % mild	76	60 (23.3)	8.1	6/6 (2.3)	51.3 % mild
										9.2 % severe
					10.3 % severe					
Back pain	59	49 (12.4)	6.4	None	40.7 % mild	71	54 (20.9)	7.6	None	29.6 % mild
										14.1 % severe
					11.9 % severe					
Dizziness	31	27 (6.8)	3.2	None	67.7 % mild	72	61 (23.6)	7.5	2/2 (0.8)	72.2 % mild
										2.8 % severe
					None severe					
Constipation	29	26 (6.6)	2.9	None	79.3 % mild	68	59 (22.9)	7.0	None	52.9 % mild
										4.4 % severe
					None severe					
Insomnia	52	45 (11.4)	5.5	1/1 (0.3)	50.0 % mild	66	56 (21.7)	7.0	None	66.7 % mild
										9.1 % severe
					3.8 % severe					
Pain in extremity	23	20 (5.1)	2.5	None	47.8 % mild	66	47 (18.2)	7.0	None	51.5 % mild
										7.6 % severe
					4.3 % severe					
Contusion	27	12 (3.0)	2.9	None	63.0 % mild	54	29 (11.2)	5.7	None	61.1 % mild
										1.9 % severe
					None severe					
Upper respiratory tract infection	8	7 (1.8)	0.9	None	62.5 % mild	51	39 (15.1)	5.4	None	52.9 % mild
										2.0 % severe
					None severe					
Weight decreased	52	43 (10.9)	5.4	None	69.2 % mild	26	24 (9.3)	2.8	None	53.8 % mild
										7.7 % severe
					1.9 % severe					

*AE* adverse event, *ASR* application site reaction

^a^All events are classified as mild, moderate or severe and total 100 %

^b^Total not equal to 100 % as six events not classified as mild, moderate or severe

^c^MedDRA high-level term, application and instillation site reactions

Overall, serious AEs occurred across system organ classes in both studies with no obvious grouping or trend. During study SP516, 148 patients (37 %) reported a total of 273 serious AEs which included 17 that were associated with the death of 15 patients (4 %), while in study SP715, 165 patients (64 %) reported a total of 437 serious AEs including 29 that led to the death of 28 patients (11 %). All the serious AEs which led to death were judged by the investigators to be unrelated to, or unlikely to be related to, study medication except for one case of myocardial infarction in study SP516 and one case each of circulatory collapse and urosepsis in study SP715 which were judged to be possibly related to rotigotine.

The same three AEs—somnolence, fall and application site reactions (ASRs)—were the most frequently reported AEs in both studies (Table 2), although there were some variations between the studies in the incidence of other common AEs. For AEs in general, most occurrences of somnolence, fall and ASRs were mild or moderate in intensity although 17 % of falls in study SP516 were recorded as severe (Table 2). Four falls by four patients (1 %) in this study were considered to be serious, while in study SP715 14 falls by 13 patients (5 %) were considered to be serious. There were no serious cases of somnolence in either study. ASRs occurred with relatively high frequency in both studies (Table 2) and of all AEs led to the greatest number of discontinuations [by 14 subjects (3.5 %)] in study SP516 (Table 2). In study SP516, the mean time from start of treatment to the first onset of an ASR was 110 (±158) days; two were serious, seven severe and 72 % had resolved by study completion. In study SP715, the mean time from start of treatment to the first onset of an ASR was 228 (±370) days; none was severe or serious; and 83 % had resolved by study completion. Analysis of the exposure-adjusted incidence (percentage per patient-year) of AEs by rotigotine dose at AE onset (Table 3) revealed that, in both studies, there was a trend toward a decreased exposure-adjusted incidence of ASRs with increasing rotigotine dose. This was true also of nausea (Table 3) though no particular trend could be identified for other common AEs. Absolute incidence of nausea was highest in the first year of open-label treatment than in all subsequent study years: in study SP516, nausea was reported by 46 patients (12 %) in year 1, five patients (2 %) in year 2, four patients (2 %) in year 3, and zero in year 4. In study SP715, nausea was reported by 44 patients (17 %) in year 1, nine patients (4 %) in each of years 2 and 3, two patients (1 %) in year 4, four patients (3 %) in year 5, and two patients (3 %) in year 6.

**Table 3:** Exposure-adjusted incidence (percentage per patient-year) of selected AEs (typical dopaminergic AEs and ASRs) by rotigotine dose at AE onset (safety set) in studies SP516 and SP715

Preferred term	Studies	Rotigotine dose						
		4 mg/24 h (235^a^/255^b^)	6 mg/24 h (859^a^/422^b^)	8 mg/24 h (1,292^a^/1,029^b^)	10 mg/24 h (1,430^a^/1,146^b^)	12 mg/24 h (3,399^a^/1,495^b^)	14 mg/24 h (2,046^a^/1,541^b^)	16 mg/24 h (2,010^a^/5252^b^)
Somnolence	SP516	18.8	11.4	32.7	11.5	16.9	20.3	17.4
	SP715	30.7	19.6	33.4	27.7	27.2	23.5	16.7
Fall	SP516	37.6	28.5	14.0	22.0	16.1	8.6	8.7
	SP715	30.7	21.0	40.8	24.3	24.4	8.8	18.5
ASR^c^	SP516	145.9	31.3	23.3	16.8	8.8	6.2	9.4
	SP715	86.9	21.0	23.2	11.7	17.3	1.8	4.2
Peripheral edema	SP516	4.7	8.5	1.2	2.1	7.2	3.1	3.2
	SP715	15.3	14.0	19.5	13.4	10.2	10.6	7.8
Nausea	SP516	61.2	17.1	21.0	8.4	4.0	3.1	1.8
	SP715	122.6	14.0	15.8	10.9	4.2	2.9	4.8
Dyskinesia	SP516	32.9	31.3	15.2	7.3	6.4	7.0	4.8
	SP715	10.2	5.6	5.6	6.7	2.5	2.9	1.2
Hallucination	SP516	4.7	5.7	2.3	6.3	2.4	7.0	3.7
	SP715	10.2	9.8	7.4	11.7	9.2	5.3	6.0
Insomnia	SP516	18.8	14.2	7.0	4.2	6.4	7.0	3.4
	SP715	15.3	1.4	11.1	7.5	7.4	4.7	7.2

*AE* adverse event, *ASR* application site reaction, *PM* sum of patient months

^a^PM value of SP715 study

^b^PM value of SP516 study

^c^MedDRA high-level term, application and instillation site reactions

In study SP516, 25 AEs in 23 patients who discontinued (nine of ASRs, four of hallucination, and one each of confusional state, delirium, pathological gambling, sleep attacks, nausea, PD, vomiting, rash with pruritis, contact dermatitis, severe skin reaction, emotional distress and erythema) were considered to be very probably related to study medication. In study SP715, only three of the AEs that led to discontinuation (hallucination, dementia and itching at application site) were considered to be very probably related to study medication.

Hallucinations and compulsive behaviors are typical side-effects of dopaminergic agonists. Indeed, hallucination led to the greatest number of discontinuations [six subjects (2 %); Table 2] in study SP715. All but seven of the 76 cases of hallucination in this study (Table 2) were mild or moderate in intensity and eight cases in eight patients (3 %) were considered to be serious. Of the 39 reported cases of hallucination in study SP516 (Table 2), all but four were mild or moderate in intensity and four cases in four patients (1 %) were considered to be serious. Of 42 AEs indicative of impulsive-compulsive behavior reported by 22 patients (6 %) in study SP516, 25 [in 16 subjects (4 %)] were assessed by the investigators as being probably or highly probably related to the trial medication. Two patients (0.5 %) discontinued the study as a result of these behaviors and four others (1 %) had their dose reduced; in all four, the AE was resolved at the end of the study. In study SP715, a total of 28 AEs indicative of impulsive-compulsive behavior were reported by 21 patients (8 %). Of these, 15 [in 10 subjects (4 %)] were assessed as being probably or highly probably related to the trial medication. Three patients (1 %) discontinued as a result of these AEs and four others (2 %) had their dose reduced; in all but one of these four patients, the AE was resolved at the end of the study. With the exception of one case of worsening of a gambling addiction, one case of pathological gambling and one case of obsessive compulsive symptoms in study SP516, and two cases of compulsive gambling in study SP715, all the AEs indicative of impulsive-compulsive behavior were judged to be not serious and almost all were mild or moderate in intensity. In addition, there was no notable variation in the incidence of psychiatric AEs, including hallucination, insomnia, confusional state, depression, anxiety, abnormal dreams and nightmare, by year of open-label treatment in either study.

The majority of patients (69 % in study SP516 and 80 % in study SP715) developed dyskinesias during the open-label extension studies (according to AE reporting or UPDRS part IV item “What proportion of the waking day are dyskinesias present?”). Across both studies, dyskinesias occurred with an incidence of 4–8 % per patient-year.

The mean ESS score increased from 7.1 (±4.5) at double-blind baseline to 8.4 (±5.2) at EoT in study SP516 and from 7.8 (±4.3) at double-blind baseline to 10.0 (±5.4) at EoT in study SP715. Except for some minor changes in hematocrit, hemoglobin and red blood cell count in study SP715, there were no clinically relevant changes in vital signs or laboratory parameters in either study.

### Efficacy

At double-blind baseline, 59 and 66 % of patients in studies SP516 and SP715 were Hoehn and Yahr stage II, respectively. By EoT, these proportions had decreased to 37 and 35 %, respectively. Concomitantly, 2 and 3 % of patients in studies SP516 and SP715 were Hoehn and Yahr stage IV, respectively, at baseline, increasing to 10 and 20 % at EoT. These data demonstrate continuing disease progression over the course of both studies.

Over the open-label titration periods of studies SP516 and SP715, the mean UPDRS part II score (ADLs) improved relative to double-blind baseline by 4.5 and 4.9 points, respectively (Figs. 2, 3). Scores then gradually increased (indicative of deterioration in ADLs) over the maintenance periods of both studies. By EoT of study SP516, the mean UPDRS part II score was still close to its baseline value (+0.8 points; Fig. 2), while in study SP715 it was 4.1 points higher (Fig. 3).

In study SP516, mean UPDRS part III scores (motor function) improved from double-blind baseline by 10.1 points during titration, then gradually declined, but were still improved relative to baseline by 2.8 points at EoT (Fig. 2). In study SP715, they declined from an initial 11.4-point improvement at the end of the titration period to baseline values (−0.2 points) at EoT (Fig. 3).

At the end of the titration periods of studies SP516 and SP715, 71 and 74 % of all patients were classified as responders on the UPDRS parts II and III sum score, respectively. Consistent with the individual UPDRS parts II and III scores, responder rates in both studies decreased over time but, at EoT, 36 % of patients in study SP516 and 24 % of patients in study SP715 were still classified as responders.

At the first visit of the maintenance periods of studies SP516 and SP715, UPDRS part IV item “What proportion of the waking day is the subject ‘OFF’ on average?” was improved relative to baseline (Visit 2 of the double-blind study) by 0.8 and 0.9 points, respectively. This improvement was maintained with little variation over the course of the maintenance periods, declining slightly to 0.4 point at EoT of study SP516 and 0.5 point at EoT of study SP715. In study SP516, a total of ten patients (3 %) spent none of their waking day in the “OFF” state at baseline (Visit 2 of the double-blind study), while at EoT this had increased to 34 patients (10 %). In study SP715, a total of 12 patients (5 %) spent none of their waking day in the “OFF” state at baseline (Visit 1 of the open-label study), while at EoT this had increased to 32 patients (12 %).

According to the mean CGI score, which was unchanged from its double-blind baseline value of 4.1 at the end of open-label treatment in SP516, there was no change in the severity of patients’ PD after 4 years of open-label rotigotine treatment. In SP715, there was a slight increase in disease severity over the 6 years of the study, as shown by an increase in mean CGI score from 3.9 at double-blind baseline to 4.1 at the end of open-label treatment.

## Discussion

In the open-label follow-up of the CLEOPATRA-PD study, 48 % of patients completed 4 years of treatment while 20 % withdrew due to AEs. In the open-label follow-up of the PREFER study, 45 % of patients completed 6 years of treatment while 28 % withdrew due to AEs. Rotigotine was well tolerated in both studies and AEs were generally mild or moderate in intensity. The two studies had similar AE profiles, with mainly typical dopaminergic effects such as somnolence, insomnia, dyskinesias, hallucinations and nausea. Indeed, together with fall and ASRs, somnolence was one of the most frequently reported AEs in both studies, but almost all cases were mild in intensity and none were serious. With the exception of ASRs, a known side-effect of the rotigotine patch, the AE profiles were similar to those observed in previous long-term studies of other dopaminergic agonists (Rascol et al. 2000; Holloway et al. 2004; Möller et al. 2005; Parkinson Study Group CALM Cohort Investigators 2009). Compulsive behaviors are known to be a typical side-effect of dopaminergic agonists but their overall incidence in these studies was low (6 % in study SP516 and 8 % in study SP715). Moreover, except for one case of worsening of a gambling addiction, one case of pathological gambling and one case of obsessive compulsive symptoms in study SP516, and two cases of compulsive gambling in study SP715, none of the AEs indicative of impulsive-compulsive behavior were serious. In addition, they were almost all mild or moderate in intensity.

The spectrum of AEs reported in the open-label extensions was similar to those in the preceding double-blind studies. However, some AEs occurred with higher frequency in the open-label study compared with the double-blind study. For example, of those AEs in study SP715 judged by the investigator as being possibly related to the study drug, fall and contusion were among the most common AEs in the open-label extension but not in the preceding double-blind study. In study SP516, insomnia, one of the AEs judged to be possibly related to the study drug, occurred with higher incidence in the open-label study compared with the double-blind one. It is not clear whether this indicates that some AEs occur only with prolonged use of rotigotine, or whether this is simply the consequence of patients’ comorbidities and age, the addition of other medications and natural disease progression over the long period of follow-up. There were also AEs that occurred less frequently in the open-label compared with the double-blind studies. For example, the incidence of nausea was 7 and 10 % per patient-year in studies SP516 and SP715, respectively, compared with an absolute incidence of 17 % in CLEOPATRA-PD and 24–28 % in PREFER. In addition, the absolute incidence of nausea was highest in the first year of open-label treatment than in all subsequent study years and its exposure-adjusted incidence was higher at lower doses of rotigotine. As these lower doses tended to be the transient titration doses, this could suggest that nausea resolves with continued use of rotigotine. It cannot be excluded that the use of anti-emetics may have contributed some part to this reduction over time. However, in study SP516, only 11 patients took anti-emetics, so it is unlikely that this was responsible for the decrease in the incidence over time. In study SP715, 28 patients took anti-emetics, but the exact contribution to reduction in nausea cannot be accurately assessed as it is not known how many patients who experienced nausea in year one subsequently discontinued for this or other reasons. These observations may also be the result of the selective drop-out of patients with nausea or the failure to enroll patients with nausea into the open-label studies. However, only two rotigotine-treated patients (1 %) discontinued CLEOPATRA-PD and eight rotigotine-treated patients (3 %) discontinued PREFER due to nausea. In addition, 92 and 99 % of those who completed the CLEOPATRA-PD and PREFER studies, respectively, entered the open-label extensions. Overall 5 % of subjects randomized to rotigotine in CLEOPATRA-PD withdrew due to AEs and 1 % due to inefficacy, while in PREFER, 17 % of those randomized to rotigotine withdrew (or were excluded from the intent-to treat population) due to AEs and 5 % due to inefficacy. Hence, it is doubtful that the patient composition of the open-label extension studies has been biased by any pre-selection on the basis of response to rotigotine in the preceding double-blind studies.

ASRs are associated with the rotigotine transdermal system, and occurred with an absolute incidence of 26 % in study SP516 and 33 % in study SP715, compared with approximately 18 % in CLEOPATRA-PD (Poewe et al. 2007) and 36 or 46 % depending on rotigotine dose in PREFER (LeWitt et al. 2007). Most ASRs in the open-label studies were also reported at the lower doses of rotigotine as seen for nausea. With discontinuation rates due to ASRs of 3 and 2 % among the rotigotine-treated patients in CLEOPATRA-PD and PREFER, respectively, again this indicates that there has been no pre-selection of patients not suffering from ASRs. These results suggest that ASRs, like nausea, may resolve with continued use. The observed ASRs did not appear to present any long-term safety or tolerability issues as they were mostly mild; none were serious and they were associated with only a few discontinuations. Only 14 patients (4 %) discontinued prematurely due to ASRs in SP516, while this was the case for only one patient (0.4 %) in study SP715. Overall rates of discontinuation due to AEs in these studies (20 and 28 % of subjects in studies SP516 and SP715, respectively) were similar to those in long-term studies with the oral dopaminergic agonists, pramipexole and ropinirole, in both early and advanced PD (Rascol et al. 2000; Holloway et al. 2004; Hauser et al. 2007; Parkinson Study Group CALM Cohort Investigators 2009). Since ASRs are not a factor for oral drugs, the comparable rate of drug discontinuation in all of the studies suggests that there are no major tolerability issues specifically associated with the use of the transdermal patch.

The occurrence of dyskinesias is of interest in long-term studies such as those described here. Even though dyskinesia is associated with long-term treatment with l-dopa, adjunctive therapy with l-dopa is eventually needed in almost all patients and it is unsurprising to find that 70 % (study SP516) and 80 % (study SP715) of patients experienced dyskinesias in these long-term studies. It is of note that these rates are significantly higher than in the preceding double-blind trials (12 % for subjects randomized to rotigotine in CLEOPATRA-PD and 14 or 17 % depending on rotigotine dose in PREFER). The high rates seen here reflect the fact that all the patients had advanced PD and were receiving l-dopa. In contrast, in a double-blind, randomized trial comparing pramipexole and l-dopa in patients with early PD, the incidence of dyskinesias was 10 % in the patients randomized to pramipexole (of whom 53 % required supplemental l-dopa), compared with 39 % in those randomized to l-dopa alone (Parkinson Study Group 2000).

While there was evidence for disease progression in patients who participated in Studies SP715 and SP516, mean UPDRS part II (ADL) scores remained improved relative to double-blind baseline for approximately 2 and 2.5 years, respectively, and patients did not return to their double-blind treatment baselines for their “ON”-rated UPDRS part III motor scores for up to 5 years. Thus, motor function, as measured using the UPDRS part III, remained improved relative to double-blind baseline for the duration of both open-label extension studies. Moreover, there was no indication of any change in therapeutic efficacy over time. It should be noted that the treating physicians had the option of adding to the anti-PD medication regimen used in the preceding CLEOPATRA-PD and PREFER double-blind, placebo-controlled studies and an average of 17 % of study participants were receiving another dopaminergic agonist in addition to rotigotine. Moreover, the dose of concomitant l-dopa was seen to increase over time in both long-term studies. This combination anti-PD treatment regimen may have contributed to both the efficacy and AE outcomes in the two extension studies reported here.

It should also be noted that there is inconsistency in the measurement of “OFF” time between the long-term studies and their respective preceding double-blind studies (Poewe et al. 2007; LeWitt et al. 2007) in which “OFF” time (in each 30-min interval during a 24-h day) was recorded in a patient diary. In the CLEOPATRA-PD study, the absolute mean change in daily “OFF” time for the rotigotine group between baseline and the end of the maintenance period was −1.6 h compared with placebo (Poewe et al. 2007). In the PREFER study, the absolute mean change in daily “OFF” time between baseline and the end of the maintenance period was −2.7 h for the 8 mg/24 h rotigotine group and −2.1 h for the 12 mg/24 h rotigotine group, compared with −0.9 h for the placebo group (LeWitt et al. 2007). The measurement of “OFF” time using a patient diary was impractical over the long period of the open-label studies and was, therefore, measured using questions in the UPDRS part IV. Although no comparisons can be made, it is clear that even after up to 6 years of open-label treatment, rotigotine treatment was associated with an improvement in the daily proportion of time spent in the “OFF” state.

In summary, these open-label extension studies have demonstrated the safety, tolerability and efficacy of the rotigotine transdermal system, in combination with l-dopa, for advanced PD patients followed for up to 6 years.

## References

[CR1] Antonini A, Tolosa E, Mizuno Y, Yamamoto M, Poewe WH (2009). A reassessment of risks and benefits of dopamine agonists in Parkinson’s disease. Lancet Neurol.

[CR2] Boroojerdi B, Wolff HM, Braun M, Scheller DK (2010). Rotigotine transdermal patch for the treatment of Parkinson’s disease and restless legs syndrome. Drugs Today (Barc).

[CR3] Giladi N, Boroojerdi B, Korczyn AD, Burn DJ, Clarke CE, Schapira AH, SP513 investigators (2007). Rotigotine transdermal patch in early Parkinson’s disease: a randomized, double-blind, controlled study versus placebo and ropinirole. Mov Disord.

[CR4] Hauser RA, Rascol O, Korczyn AD, Jon Stoessl A, Watts RL, Poewe W, De Deyn PP, Lang AE (2007). Ten-year follow-up of Parkinson’s disease patients randomized to initial therapy with ropinirole or levodopa. Mov Disord.

[CR5] Holloway RG, Shoulson I, Fahn S (2004). Pramipexole vs levodopa as initial treatment for Parkinson disease: a 4-year randomized controlled trial. Arch Neurol.

[CR6] Jankovic J, Watts RL, Martin W, Boroojerdi B (2007). Transdermal rotigotine: double-blind, placebo-controlled trial in Parkinson disease. Arch Neurol.

[CR7] Jenner P (2005). A novel dopamine agonist for the transdermal treatment of Parkinson’s disease. Neurology.

[CR8] LeWitt PA (2008). Levodopa for the treatment of Parkinson’s disease. N Engl J Med.

[CR9] LeWitt P, Kompoliti VM (2010). Dopaminergic agonists in Parkinson’s disease. Encyclopedia of movement disorders.

[CR10] LeWitt PA, Lyons KE, Pahwa R, SP 650 Study Group (2007). Advanced Parkinson disease treated with rotigotine transdermal system: PREFER study. Neurology.

[CR11] Möller JC, Oertel WH, Köster J, Pezzoli G, Provinciali L (2005). Long-term efficacy and safety of pramipexole in advanced Parkinson’s disease: results from a European multicenter trial. Mov Disord.

[CR12] Nutt JG (2001). Motor fluctuations and dyskinesia in Parkinson’s disease. Parkinsonism Relat Disord.

[CR13] Olanow CW, Obeso JA, Stocchi F (2006). Drug insight: continuous dopaminergic stimulation in the treatment of Parkinson’s disease. Nat Clin Pract Neurol.

[CR14] Pahwa R, Poewe WH, Lyons KE, Boroojerdi B (2009). Changes in early morning motor status following adjunctive treatment of advanced Parkinson’s disease with rotigotine transdermal system in two large, placebo-controlled trials (PREFER and CLEOPATRA-PD). Mov Disord.

[CR15] Parkinson Study Group (2000). Pramipexole vs levodopa as initial treatment for Parkinson disease: a randomized controlled trial. Parkinson Study Group. JAMA.

[CR16] Parkinson Study Group (2003). A controlled trial of rotigotine monotherapy in early Parkinson’s disease. Arch Neurol.

[CR17] Parkinson Study Group CALM Cohort Investigators (2009). Long-term effect of initiating pramipexole vs levodopa in early Parkinson disease. Arch Neurol.

[CR18] Pham DQ, Nogid A (2008). Rotigotine transdermal system for the treatment of Parkinson’s disease. Clin Ther.

[CR19] Poewe WH, Rascol O, Quinn N, Tolosa E, Oertel WH, Martignoni E, Rupp M, Boroojerdi B, SP 515 Investigators (2007). Efficacy of pramipexole and transdermal rotigotine in advanced Parkinson’s disease: a double-blind, double-dummy, randomised controlled trial. Lancet Neurol.

[CR20] Rascol O, Perez-Lloret S (2009). Rotigotine transdermal delivery for the treatment of Parkinson’s disease. Expert Opin Pharmacother.

[CR21] Rascol O, Brooks DJ, Korczyn AD, De Deyn PP, Clarke CE, Lang AE (2000). A five-year study of the incidence of dyskinesia in patients with early Parkinson’s disease who were treated with ropinirole or levodopa. 056 Study Group. N Engl J Med.

[CR22] Trenkwalder C, Kies B, Rudzinska M (2011). Rotigotine effects on early morning motor function and sleep in Parkinson’s disease: a double-blind, randomized, placebo-controlled study (RECOVER). Mov Disord.

[CR23] Watts RL, Jankovic J, Waters C, Rajput A, Boroojerdi B, Rao J (2007). Randomized, blind, controlled trial of transdermal rotigotine in early Parkinson disease. Neurology.

